# Cloning Should Be Simple: *Escherichia coli* DH5α-Mediated Assembly of Multiple DNA Fragments with Short End Homologies

**DOI:** 10.1371/journal.pone.0137466

**Published:** 2015-09-08

**Authors:** Maxim Kostylev, Anne E. Otwell, Ruth E. Richardson, Yo Suzuki

**Affiliations:** 1 Department of Synthetic Biology and Bioenergy, J. Craig Venter Institute, La Jolla, California, United States of America; 2 Department of Microbiology, Cornell University, Ithaca, New York, United States of America; 3 School of Civil and Environmental Engineering, Cornell University, Ithaca, New York, United States of America; Imperial College London, UNITED KINGDOM

## Abstract

Numerous DNA assembly technologies exist for generating plasmids for biological studies. Many procedures require complex *in vitro* or *in vivo* assembly reactions followed by plasmid propagation in recombination-impaired *Escherichia coli* strains such as DH5α, which are optimal for stable amplification of the DNA materials. Here we show that despite its utility as a cloning strain, DH5α retains sufficient recombinase activity to assemble up to six double-stranded DNA fragments ranging in size from 150 bp to at least 7 kb into plasmids *in vivo*. This process also requires surprisingly small amounts of DNA, potentially obviating the need for upstream assembly processes associated with most common applications of DNA assembly. We demonstrate the application of this process in cloning of various DNA fragments including synthetic genes, preparation of knockout constructs, and incorporation of guide RNA sequences in constructs for clustered regularly interspaced short palindromic repeats (CRISPR) genome editing. This consolidated process for assembly and amplification in a widely available strain of *E*. *coli* may enable productivity gain across disciplines involving recombinant DNA work.

## Introduction

Recombinant DNA technologies have been critical for driving biotechnological advances and facilitating studies aimed at understanding basic biological principles. Despite its limitations, restriction digestion- and ligation-based cloning is still widely used to generate DNA constructs for a variety of molecular biology applications. At the same time, techniques for the seamless assembly of DNA have been rapidly expanding, enabling more precise genetic manipulation in synthetic biology and metabolic engineering. Many of these methods rely on the annealing of strands from neighboring DNA fragments, allowing for assembly of fragments with overlapping ends. Both *in vitro* [1–10] and *in vivo* [11–21] techniques have been developed for this purpose.

A particular advantage of *in vivo* techniques is that they do not require externally added purified enzymes. The yeast *Saccharomyces cerevisiae* is used as a host organism for *in vivo* DNA assembly due to its ability to efficiently repair double-strand breaks. Multiple linear DNA fragments can be taken up and accurately assembled via homologous recombination in yeast [11–15]. However, relative to *S*. *cerevisiae*, *Escherichia coli* offers a number of strengths as a host organism for *in vivo* DNA assembly including faster growth rates, higher plasmid yields, and greater transformation efficiency. A number of studies have led to the development of *in vivo* DNA assembly methods in *E*. *coli*, primarily utilizing the RecA-independent λ phage- and Rac prophage-based systems (λ Red and RecET, respectively) [16–18]. *E*. *coli* also has been shown to contain endogenous RecA-independent homologous recombination activities, but the mechanisms remain to be fully characterized [2,21–24].

One of the most common laboratory *E*. *coli* strains used to maintain and amplify small plasmid DNA is K-12 derived DH5α. Typical DNA assembly and cloning procedures involve as their last step transformation of the constructed plasmid into competent DH5α cells. Even for λ Red- and RecET-based methods, it is recommended that the *in vivo* assembled plasmid be transferred into a cloning strain such as DH5α to ensure stability of the DNA product [18]. It has been previously shown that DH5α cells have some ability to recombine *in vivo* heterologous DNA fragments with homologous ends, albeit at relatively low efficiency [19–21,25]. We therefore explored the ability of this strain to recombine DNA for the purpose of simplifying basic DNA cloning and multi-fragment assembly.

**Fig 1:**
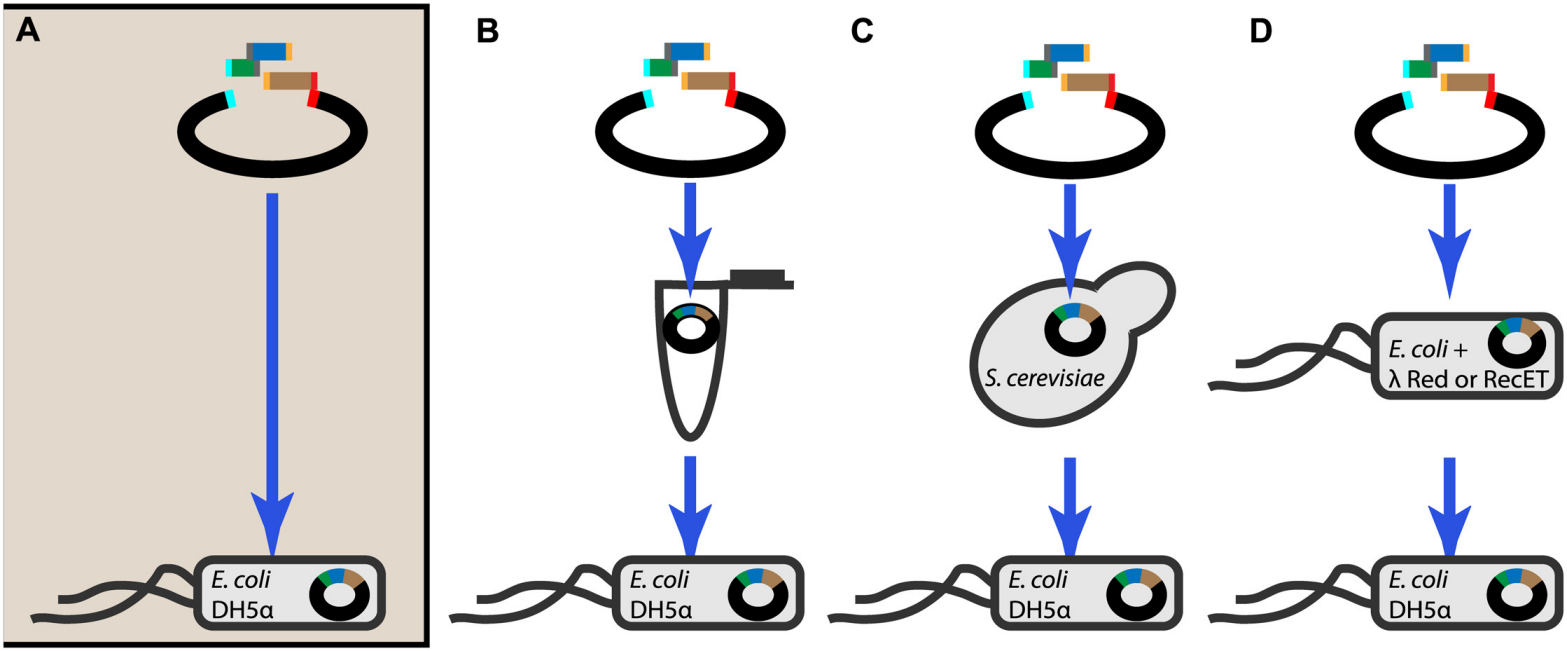
*In vivo* DNA assembly and cloning in *E*. *coli* DH5α. (**A**) *E*. *coli* DH5α-mediated DNA assembly involves only two basic steps: 1) preparation of fragments with homologous ends and 2) introduction of the fragments into competent cells. This approach minimizes the time and reagents required for DNA assembly in comparison to other common methods, which contain a separate assembly step before the introduction of the constructed plasmid into a recombination-impaired cloning strain such as DH5α (**B-D**). Assembly is typically carried out either with added enzymes *in vitro* (**B**), or *in vivo*, using as a host *S*. *cerevisiae* (**C**) or specialized *E*. *coli* strains expressing the λ Red or RecET phage-based systems (**D**).

Our scheme for *E*. *coli* DH5α-mediated DNA assembly involves only two basic steps: preparation of DNA fragments to be assembled and introduction of the fragments into competent cells (Fig 1). When PCR is used to generate these fragments from plasmids that share the marker used for the final transformant selection, the PCR templates can contribute to false positives where transformants contain no assembled products. These templates can be conveniently removed using DpnI restriction enzyme, which specifically destroys *E*. *coli*-derived templates at methylated GATC sequences while not affecting non-methylated PCR fragments. After the fragments are prepared, they are directly introduced into DH5α cells. DNA amplification, DpnI digestion, and transformation can be completed in one day. This is a simple and rapid DNA assembly technique that can be employed for a variety of applications.

## Materials and Methods

### Bacterial strains

The following commercial products were used: Max Efficiency DH5α (Life Technologies, Carlsbad, CA; chemically competent, ~10^9^ colony-forming unit or CFU / μg pUC19), High Efficiency NEB 5-alpha (New England Biolabs, Ipswich, MA; chemically competent, CFU ~10^9^/μg pUC19), and NEB 5-alpha Electrocompetent *E*. *coli* (New England Biolabs, CFU ~10^10^/μg pUC19).

### DNA

Original pUC19 and pBR322 vectors were used for most assemblies. For the cloning of cellulase genes, a vector derived from pYOGM081 [26] was used. The following modifications were made (T. Hanly, M.K., and Y.S., unpublished result). The *att* sites for Gateway cloning (Life Technologies) were removed and *GAL1-10* promoter for expressing an exogenous gene was replaced with *ENO1* promoter, followed by sequences encoding the 19-amino acid signal sequence of *S*. *cerevisiae* mating factor alpha 1, Ala-Gly dipeptide, human influenza hemagglutinin epitope tag, 17-amino acid Gly-Ser linker (codon-optimized for *S*. *cerevisiae*; DNA 2.0, Inc., Menlo Park, CA), Ser-Thr linker (codon-optimized for *S*. *cerevisiae*; DNA 2.0, Inc.), and dockerin from *Ruminococcus flavefaciens cel44A* gene (codon-optimized for *S*. *cerevisiae*; DNA 2.0, Inc.). Between the sequences for the Gly-Ser linker and the Ser-Thr linker is the cellulase insertion site. For gRNA plasmid alteration experiments, p426-SNR52p-gRNA.CAN1.Y-SUP4t (6.3 kb; [27]) and P_TRC_ gRNA pUC (3.2 kb; P. Weyman and K. Schmitz, unpublished result) plasmids were used.

Cellulase genes (S1 Table) were synthesized using BioXp 3200 system (SGI-DNA, La Jolla, CA). All the fragments contained at their 5’ and 3’ ends 40-bp homology to the insertion site of the above vector, as well as so-called Gibson ends outside of the user defined sequences. Gel-purification is recommended in the manufacturer protocol to remove intermediate by-products (SGI-DNA). To aid with high-throughput assembly of 29 constructs, we modified the protocol by replacing gel-purification with PCR amplification. The synthesized genes were PCR-amplified using the primers that matched the 5’ and 3’ homologous sequences (S2 Table) and purified using Nucleospin Gel and PCR Clean-Up kit (Macherey-Nagel, Bethlehem, PA).

The sequence encoding a 17-amino acid Gly-Ser linker and three cohesin domains of *Clostridium thermocellum cipA* gene was codon-optimized (Integrated DNA Technologies, Coralville, IA) for expression in *S*. *cerevisiae* and synthesized as 3 gBlocks fragments 966, 499, and 987 bp in length (Integrated DNA Technologies).

All DNA fragments, including the synthesized fragments, were PCR-amplified using high-fidelity DNA polymerases PrimeSTAR Max (2× Master Mix, Takara Bio, Mountain View, CA) or Q5 (Hot-Start 2× Master Mix, New England Biolabs). Primers and templates are listed in S2 Table. When applicable, PCR products were subjected to DpnI digest (New England Biolabs; CutSmart buffer was added to final concentration of 1× for all digests) for ~2 hours at 37°C. Nucleospin Gel and PCR Clean-Up kit (Macherey-Nagel) was used for PCR product purification.

### Transformation of *E*. *coli* and assembly verification

Transformation of Max Efficiency DH5α competent cells was modified from the manufacturer’s protocol as follows. 25 μl of cells were used per transformation, corresponding to one fourth of the recommended cell volume. Cells were transferred to 2 ml polypropylene tubes (Axygen, Union City, CA). DNA was diluted and mixed in Milli-Q purified sterile water and 2.5 μl was added per transformation. No difference in transformation efficiency was observed when the DNA was prepared in 10 mM Tris-HCl buffer, pH 8.5, with or without 1 mM EDTA. Cells and DNA were incubated on ice for 30 minutes and then placed in a 42°C water bath for 45 seconds. Following a two- to five-minute incubation on ice, 225 μl of room temperature SOC medium (Life Technologies) was added to the tubes, and the cells were allowed to recover at 37°C with shaking at 250 rpm for one hour. Cells were then plated on LB-agar plates with appropriate antibiotics (100 μg/ml ampicillin, 60μg/ml kanamycin) and X-gal/IPTG, when applicable. Plates were incubated at 37°C overnight. Transformation of NEB 5-alpha chemically competent cells was the same as above with the following modifications. 25 μl of cells corresponded to half of the recommended cell volume per transformation. The cells were placed at 42°C for 30 seconds and were allowed to recover in 450 μl of SOC medium. Transformation of NEB 5-alpha electrocompetent cells was performed following the manufacturer’s protocol.

For p426-SNR52p-gRNA.CAN1.Y-SUP4t self-closure experiments 1 ng of the original gRNA plasmid was used as template DNA in a 50-μl PCR reaction. PrimeSTAR Max polymerase (2× Master Mix, Takara Bio) was used to generate both altered plasmids. PCR reactions were digested with DpnI for ~2 hours at 37°C and purified with Nucleospin Gel and PCR Clean-Up kit (Macherey-Nagel). 1 μl of the purified product (125–150 ng DNA) was combined with 25 μl NEB 5-alpha chemically competent cells and transformation was performed as above. For the P_TRC_ gRNA pUC alteration, PCR was carried out using 0.4 ng of the original gRNA plasmid and PrimeSTAR Max polymerase in a 20-μl reaction. PCR product was purified (without DpnI digest). When 1 μl (38 ng) of the purified product was combined with 10 μl NEB 5-alpha chemically competent cells, ~1,000 colonies formed on an ampicillin plate.

To verify correct assembly of the plasmids (not including those in plasmid-alteration experiments), colony PCR was performed with Quickload OneTaq polymerase (2× Master Mix, New England Biolabs) using primers outside of the insertion junctions (S2 Table). For Sanger sequencing, colonies were cultured in LB medium containing the appropriate antibiotics and the DNA was isolated using a miniprep kit (Qiagen, Valencia, CA).

## Results

### Single-fragment cloning

If DNA constructs can be generated and propagated in a single organism, the overall workflow in molecular biology can be considerably simplified with far-reaching impacts on scientific advances. According to published data, *E*. *coli* is able to recombine DNA fragments that share more than ~20-base end homology and the efficiency increases with increasing homology length regardless of the recombination mechanism [2,16,22,23,25,28]. We chose ~50-bp end homology for most of the experiments described here because this length is readily attainable using standard commercially available DNA primers. Reasoning that transformation efficiency may play an important role in the overall cloning efficiency, we chose commercially available, highly competent (~10^9^ colony-forming unit or CFU / μg pUC19) DH5α cells. In preliminary experiments we determined that chemically competent cells are more efficient than electrocompetent ones (Table 1).

**Table 1:** Assembly of pUC19 by different commercial strains.

*E*. *coli* strain	Expected transformation efficiency (CFU/μg pUC19)	Blue colonies per 25 μl cells^a^ ^,^ ^b^	Viable cell counts per 25 μl cells	Blue colonies per 10^9^ viable cells
Max Efficiency DH5α^TM^, chemically competent (Life Technologies)	>1 x 10^9^	821	3 × 10^9^	243
High efficiency NEB 5-alpha, chemically competent (New England Biolabs)	1–3 x 10^9^	619	2 × 10^9^	292
NEB 5-alpha, electrocompetent (New England Biolabs)	>1 x 10^10^	507	4 × 10^10^	13

^a^ 1 ng vector was used per transformation with a molar insert-to-vector ratio of 5:1.

^b^ 25 μl cells corresponds to 1/4, 1/2, and 1 recommended transformation cell volume in rows 1, 2, and 3, respectively.

**Fig 2:**
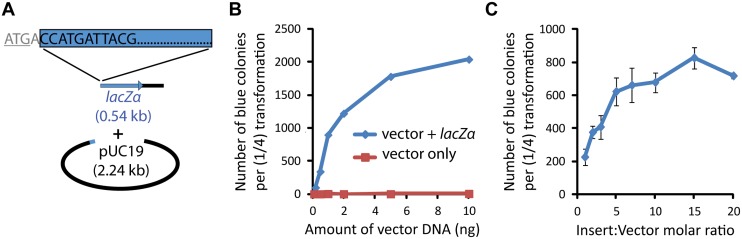
pUC19-*lacZα* assembly assay. (**A**) Two fragments were PCR-amplified from the pUC19 vector to create an efficient screen for DNA assembly capability. The smaller “insert” fragment contained the coding sequence of the *lacZα* gene starting at position five and some downstream vector sequence. The larger “vector” fragment contained the rest of the plasmid, including the Amp resistance gene (*bla*) and the origin of replication. The fragments shared 50-bp homology at both ends. (**B**) Blue colony formation as a function of DNA concentration. Very few white colonies were observed on any of the plates (S3 Table). Small numbers of blue colonies present in the vector-only transformations are indicative of the small amount of contaminating circular template pUC19 used in PCR-mediated linearization of the vector and undigested during DpnI treatment. Insert-to-vector molar ratio was maintained at 5:1, and 25 μl of cells were used, corresponding to ¼ of the recommended volume. (**C**) Effect of insert-to-vector molar ratio on assembly efficiency. The vector DNA quantity was maintained at 0.5 ng. Error bars indicate standard deviation from two independent sets of experiments.

To determine the efficiency and fidelity of DH5α-mediated *in vivo* assembly and cloning, we designed a pUC19 plasmid-based screen (Fig 2A). In this work, efficiency is defined as the number of colonies obtained per quantity of added DNA. Fidelity is defined as the presence of all introduced fragments in a generated construct. Two fragments of pUC19 were PCR-amplified to create an insert and a vector (544 bp and 2,241 bp, respectively) with 50 bp of homology at their ends. The insert contained the coding sequence of the *lacZα* gene starting at nucleotide position five, as well as some of the downstream pUC19 plasmid sequence, while the vector contained the rest of the plasmid, including the ampicillin (Amp) resistance gene (*bla*) and the origin of replication. This design was employed for two reasons. Primarily, it allowed for efficient screening of correctly assembled plasmids, identified as blue colonies on X-gal-containing agar plates (S1 Fig). In addition, it enabled us to determine whether the few colonies observed in vector-only (negative control) transformations were due to template plasmid carryover (blue colonies) or other events such as self-closure and chromosomal integration of the vector (mostly white colonies), potentially mediated by non-homologous end joining or microhomology-based recombination [29].

To determine the effect of DNA quantity on transformation efficiency, we tested a range of 0.1 to 10 ng vector DNA per ~3 × 10^9^ cells (25 μl cell suspension, corresponding to ¼ of the recommended transformation volume), maintaining an insert-to-vector molar ratio of 5:1 (Fig 2B). At low DNA concentrations, the number of colonies depended strongly on the amount of DNA added to each transformation, but the effect became less pronounced at higher DNA concentrations. Importantly, nearly all colonies were blue, indicating correct assembly of the plasmid. The few white colonies accounted for less than 1% of the total colony number (S3 Table). Negative control transformations using either the insert or the vector alone were done to quantify the amount of undigested pUC19 template DNA from DpnI digest. As expected, very few colonies were observed even at the highest DNA concentrations used (S3 Table). Altogether, the colony numbers on the negative control plates and the white colonies present on the experimental plates accounted for less than 1% of the total colony numbers. Sanger sequencing of DNA from ten colonies confirmed correct coding and junction sequences in all cases.

In some DNA assembly techniques, the molar ratios of DNA fragments can affect assembly efficiency, with an excess of small fragments leading to maximal efficiency [2,10,30]. To determine whether this is the case for the current method, we tested insert-to-vector molar ratios in the range of 1:1 to 20:1 with the pUC19 plasmid-based screen (Fig 2C). Vector DNA concentration was maintained at 0.5 ng. Increasing the insert-to-vector molar ratio resulted in an increased number of colonies, with the effect being most evident at lower ratios and hitting the point of diminishing returns around 5:1.

Transformation efficiency decreases with increasing plasmid size [31] and pUC19 is one of the smallest commonly used cloning vectors. To verify that the results obtained in the pUC19-based experiments apply to the generation of larger plasmids, we tested the ability of DH5α to assemble a 1.4-kb cellulase gene (*cbhA*) from *Aspergillus niger*, codon-optimized for *S*. *cerevisiae* [32], with a custom 7-kb high-copy vector (containing the pBluescript SK+ backbone) derived from the plasmid pYOGM081 [26]. The two fragments, designed to share 50 bp of homology at each end, were amplified using PCR. We tested 0.1–100 ng vector DNA with insert-to-vector molar ratio of 5:1 and observed a similar pattern as that seen for pUC19 (S2 Fig). The efficiency of assembly, quantified as colony number, was lower than that observed with pUC19, presumably due to the larger size of the DNA fragments. Seven to 852 colonies were obtained for the tested DNA concentrations. Thirty colonies were analyzed using colony PCR, and all showed the insert of the correct size (S3 Fig). Sanger sequencing of 20 junctions confirmed correct assembly in all but one case, where a one-base deletion was identified. This may be due to an erroneous primer having been incorporated in the PCR amplification of the fragments to be assembled.

Based on the results obtained with the *cbhA* construct assembly, we used this method to assemble a library of 29 cellulase genes into the same custom vector, for use in a different project in our group. Bacterial and fungal cellulase genes were chosen from the CAZy database (www.cazy.org), codon-optimized for yeast expression [32], and synthesized in-house using the BioXp^TM^ 3200 system (SGI-DNA, La Jolla, California). All synthesized fragments were PCR-amplified using primers that matched part of the universal sequences upstream and downstream of the gene (See Materials and Methods; S2 Table). All amplified fragments contained at each end 40-bp sequences homologous to the vector. To enable high-throughput assembly, the transformation was scaled in half (~1.5 × 10^9^ cells, corresponding to ⅛ of the recommended cell suspension volume) and carried out in PCR strips. For each assembly, 5 ng vector was mixed with 5 ng cellulase fragment, resulting in an insert-to-vector molar ratio between 5:1 and 9:1. Transformations produced four to 112 colonies per reaction (S1 Table), indicating that sequence length, composition, or structure of the insert may significantly affect assembly efficiency. We also observed a batch effect, where one group of assemblies produced substantially more colonies than the other (S1 Table, reactions 1–16 vs. 17–29). This may have been caused by slight differences between batches of cells and/or experimental techniques. One to three colonies were picked for each construct and tested using colony PCR, and the desired construct was obtained in all attempted assemblies. A great majority of tested colonies (47 out of 54) showed the insert of the expected size, and in most cases, incorrect assemblies correlated with PCR products that showed abnormalities (e.g., the presence of non-specific bands and smears; S1 Table). Altogether, our results demonstrate the utility of *E*. *coli*-mediated assembly for high-throughput cloning in an efficient and cost effective manner, requiring small amounts of DNA and minimal screening.

### Multi-fragment cloning

**Fig 3:**
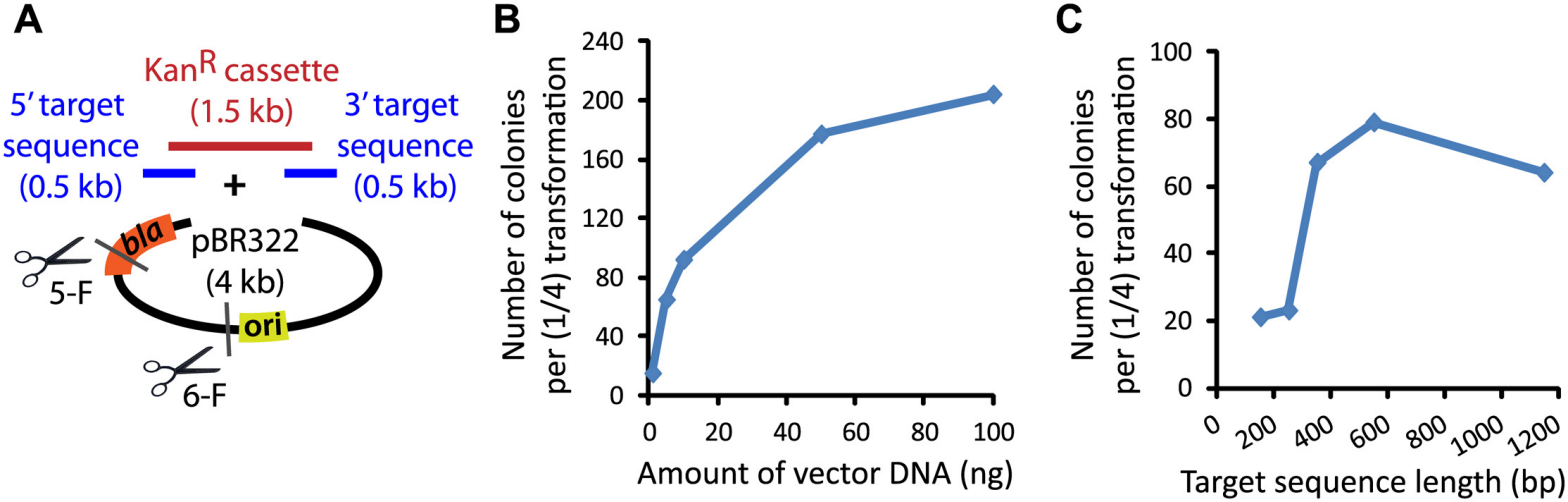
Knockout cassette assembly for the deletion of GSU 1371 from *Geobacter sulfurreducens*. (**A**) The GSU 1371 knockout construct was assembled from four fragments including 0.5-kb sequences upstream and downstream of GSU 1371, the kanamycin cassette (amplified from pET28a), and the PCR-linearized pBR322 vector. All adjacent fragments shared 50-bp end homology. In five- and six-fragment assembly experiments, a fifth site (5-F) and both the fifth and sixth sites (5-F and 6-F), respectively, were used to make additional junctions with homology for assembly. (**B**) Assembly efficiency as a function of DNA concentration was examined using plates containing both Kan and Amp. Colony PCR confirmed the correct insertion in 30/30 transformants tested (S4 Fig). (**C**) The size of the fragments upstream and downstream of GSU 1371 was varied to test the effect on DNA assembly efficiency. Reducing the length of fragments to 250 bp and less resulted in a substantially lower number of colonies. Molar ratio of insert fragments-to-vector was maintained at 5:1 in all experiments.

In many instances, it is necessary to assemble several DNA fragments, such as in the construction of fusion proteins, gene knockout cassettes, and large genes from smaller synthesized fragments. To explore whether DH5α is able to combine *in vivo* several fragments in one transformation event, we attempted the assembly of a gene knockout construct using three PCR-amplified fragments and a PCR-linearized pBR322 vector. The knockout cassette was designed to delete gene GSU 1371 from *Geobacter sulfurreducens*. 0.5-kb sequences upstream and downstream of GSU 1371 were PCR-amplified from *G*. *sulfurreducens* genomic DNA, and the kanamycin (Kan) cassette was amplified from the pET28a vector (Fig 3A). An important advantage of knockout cassette assembly is the option to apply double selection to the transformed colonies (when the marker for knockout, in addition to the one for plasmid maintenance, is functional in *E*. *coli*), which promotes high fidelity of the obtained constructs. Furthermore, when using double selection, we found it unnecessary to remove residual template vector DNA with DpnI (S4 Table), which further simplifies the overall assembly protocol.

Similar to the single-fragment experiments, we tested the effect of added DNA quantity, ranging from 1 to 100 ng, on the efficiency of multi-fragment assemblies (quantified as colony numbers obtained per transformation; Fig 3B). The molar ratios of all “insert” fragments with respect to the vector were maintained at 5:1. As for single-fragment cloning, increasing the amount of added DNA resulted in an increased number of colonies, with the effect more pronounced at low DNA concentrations. PCR analysis of 30 colonies confirmed the presence of an insert of the correct size (S4 Fig), and restriction analysis of five colonies resulted in the expected pattern in all cases. The assembled knockout cassette was successfully used to remove GSU 1371 from *G*. *sulfurreducens* (A.E.O and R.E.R., unpublished result).

It is likely that sizes of DNA fragments affect efficiency and accuracy of assembly. In addition to absolute sizes of fragments, relative sizes or differences in size among the fragments may affect DNA assembly. To evaluate the effect of fragment size on DNA assembly efficiency, we varied the length of the fragments upstream and downstream of GSU 1371 (targeting fragments) from 150 to 1,150 bp (including the overlap regions), working with 10 ng vector DNA and insert-to-vector molar ratios of 5:1 (Fig 3C). Assemblies with 150- and 250-bp targeting fragments resulted in significantly reduced colony numbers compared to those with targeting sequences 350 bp and longer. Since the efficiency of cellular entry is not expected to be reduced for smaller DNA fragments [31], our results suggest that processes subsequent to this step are unfavorable to incorporating short fragments. It is possible that such fragments are more easily degraded by exonuclease action before they are able to recombine with a neighboring fragment [23].

While double selection is an inherent advantage in the assembly of gene knockout constructs, this option is not available in many other DNA assembly applications. To compare the effect of single and double selection on fidelity, we repeated the knockout construct assembly using PCR-amplified vector DNA that had been treated with DpnI, and plated transformed cells on medium containing either both Kan and Amp or only Amp (matching the resistance on vector). Fidelity of the constructs was quantified as the fraction of colonies showing a correct band in colony PCR. Selection on Amp alone resulted in about twice as many colonies as that on both antibiotics, while fidelity of the assembled constructs decreased to 77% (27/35 colonies; S5 Table and S5 Fig). We observed similar results in the assembly of another construct. Codon-optimized scaffoldin gene *cipA* from *Clostridium thermocellum* was synthesized in three fragments, which were then used as templates to generate three PCR fragments. These were assembled into a pUC19 vector and selected on Amp plates. Colony numbers and fidelity are comparable to those obtained with the knockout construct assembly (S6 Table and S6 Fig), suggesting that the presented method can be used successfully for a variety of multi-fragment assemblies.

To explore the limits of the number of fragments that can be assembled in *E*. *coli*, we split the pBR322 vector in either two or three fragments and repeated the knockout construct assemblies, now with a total of five or six fragments (Fig 3A). For the five-fragment assembly, the vector was split within the Amp resistance gene, generating 765- and 3,320-bp fragments with 50-bp overlaps. For the six-fragment assembly, the 3,320 bp fragment was further split, generating 1,567- and 1,803-bp fragments. For both assemblies, all of the fragments were mixed in equimolar ratios such that the total amount of DNA from all fragments combined was ~125 ng (~30 fmol of each fragment) per transformation reaction. Transformants were selected on plates containing either Amp alone or both Kan and Amp, and a subset of colonies was analyzed using colony PCR (S7 Table). As expected, the colony numbers decreased significantly with the increasing number of fragments. For the five-fragment assembly, 10 (Kan-Amp) and 19 (Amp) colonies were obtained, whereas the six-fragment assembly resulted in only four (Kan-Amp) and 14 (Amp) colonies. As was the case for the four-fragment assembly with double-selection, all of the tested colonies for both five- and six-fragment assemblies selected on Kan-Amp plates had the correct knockout cassette as determined using colony PCR. Fidelity dropped to ~50–75% of the tested colonies from Amp-only plates (S7 Table). Furthermore, restriction analysis performed on plasmids isolated from three colonies revealed the expected band pattern, confirming that the DH5α-mediated method is effective for multi-fragment DNA assembly.

### Plasmid alteration via self-closure

Along with the assembly of one or more fragments into a vector, it is sometimes useful to alter existing plasmids. Common applications include the introduction of a point mutation in a cloned gene or deletion of a coding or regulatory region already in a plasmid. Presently, a powerful targeted genome editing technique based on clustered regularly interspaced, short palindromic repeats (CRISPR) is emerging. The most studied and developed system uses CRISPR-associated protein 9 (Cas9) from *Streptococcus pyogenes* to create a double-stranded DNA break at a specified sequence [33]. Cas9 is targeted to a specific 20-bp sequence by a matching protospacer sequence of a guide RNA (gRNA), which is typically encoded on a plasmid. Once Cas9 and gRNA plasmids are constructed for engineering of an organism, Cas9 activity can be targeted to any new region of the genome simply by introducing an appropriate 20-bp gRNA sequence on the already constructed plasmid. Based on the demonstrated ability of DH5α to recombine DNA with homologous overlaps, we tested our method for the simple replacement of short sequences in two different plasmids for expressing gRNA (see Materials and Methods). In one case, two new plasmids were derived from a 6.3-kb plasmid p426-SNR52p-gRNA.CAN1.Y-SUP4t [27] for the engineering of previously published *S*. *cerevisiae GMToolkit*-**a** and *GMToolkit*-**α** strains [34]. One plasmid was altered to target neutral locus YGR176W while the other to target a component of the *GMToolkit* module [34] not needed in subsequent applications. In another case, we altered a sequence in a 3.2-kb plasmid, P_TRC_ gRNA pUC, for expressing gRNA in *Klebsiella pneumoniae* (P. Weyman and K. Schmitz, unpublished result) to target *fepC*, which encodes a ferric enterobactin transport ATP-binding protein [35]. In each case, the original gRNA-encoding plasmid was PCR-linearized using primers that contained the new 20-bp target sequence (S2 Table). The resultant linear products, containing ends with 25- or 20-base homologous overlaps, were introduced into *E*. *coli*. Two colonies were picked for each of the p426-SNR53p-gRNA-based plasmids, and the correct new target sequence was confirmed in all using Sanger sequencing. P_TRC_ gRNA pUC-based plasmids were purified from five colonies. All plasmids were of the expected size for a correctly recircularized plasmid, as assayed using agarose gel electrophoresis, and all plasmids contained the intended sequence in the gRNA region based on data from Sanger sequencing. The success of these experiments indicates that *E*. *coli*-mediated assembly can be readily integrated in existing CRISPR genome editing protocols for the quick alteration of constructed gRNA plasmids.

## Discussion

Reducing the number of steps in DNA assembly has several important advantages. It can minimize the time and cost associated with the process, as well as with training for the technique and troubleshooting (because the sources and chance of errors can be simultaneously reduced). It can also promote rapid adoption of the developed methods by the research community. To this end, we demonstrate what we believe to be the simplest and fastest method for the accurate assembly of DNA ranging from 150 to several thousand base pairs.

Bubeck et al. previously showed that *E*. *coli* DH5α has the ability to recombine linear DNA fragments sharing short homologous ends, thus making it possible to carry out simple *in vivo* fragment assembly [25]. However, the utility of this earlier published effort was limited by the cloning efficiency and fidelity. The low efficiency was likely due to the CaCl_2_ method with relatively low cell competence (~10^6^ CFU / μg pBluescript SK- [25]). The perceived low fidelity was probably due to circular vector carryover, and this is indicated by high numbers of colonies observed in negative control vector-only transformations. To improve on these limitations, we used highly competent cells (~10^9^ CFU / μg pUC19) and linearized the vector using PCR followed by a DpnI digest. Using highly competent cells helps ensure that a sufficient number of cells take up all of the DNA fragments that are required for correct assembly even at low concentrations of DNA. Compared to a traditional approach of vector linearization using restriction digest, PCR-mediated amplification of a linearized vector from a minute quantity of template plasmid DNA followed by the elimination of the methylated template ensures a purer sample of the linearized vector with little circular vector DNA. Our results show that for single-fragment cloning, using small amounts of DNA (<1–10 ng), it is possible to obtain hundreds to thousands of colonies, nearly all of which carry the correctly assembled recombinant plasmid. In subsequent DNA assemblies in our group, we have found that for single-fragment cloning, the DpnI digest of template plasmid DNA (used in sub-nanogram quantities for PCR) can be optionally skipped with only a small reduction in the fidelity due to the high cloning efficiency of such constructs. We also expanded the applications of DH5α-mediated cloning to multi-fragment *in vivo* DNA assembly and to the alteration of existing plasmids, in particular for use with CRISPR-Cas9 genome editing protocols.

An approach very similar to ours, describing DH5α-mediated DNA assembly and cloning, was published [21] during the writing stage of this manuscript. In that study, up to three DNA fragments, prepared using PCR, were introduced into chemically-competent cells and the majority of the selected colonies carried the desired DNA construct. However, the cloning efficiency was up to 100 times lower than that reported in our experiments. For example, in single-fragment cloning experiments using 25 ng of pUC19 and molar insert-to-vector ratio of 2:1, only ~40 colonies were reported. Importantly, the higher cloning efficiencies observed with our method make it possible to assemble more fragments (up to six in our experiments) and simplify high-throughput cloning due to substantially lower requirements for DNA concentrations. Based on the experimental descriptions provided in the other study, we believe that several factors may explain the discrepancy. The authors used 30 bp homologous overlaps in most of their experiments, while we designed 40–50 bp overlaps for all of the reported assemblies. The transformation efficiency of the competent cells (prepared in-house using rubidium chloride method [36]) was not stated and it is possible that it was substantially lower than that of the commercial cells used in our work. Finally, the differences in the transformation protocols may also affect the overall cloning efficiency in our methods, although it is likely that the protocols should be optimized for the specific cells used. Three other efforts, relying on the same basic principle of introducing PCR-amplified DNA products into *E*. *coli* had been previously published [19,20,30]. In these studies the cloning efficiency is also relatively low and in two of them [19,20] the proposed basis of assembly was attributed to the annealing of complementary single stranded DNA overhangs common to PCR products. However, in the study by Jacobus and Gross [21], this hypothesis is disproven in cloning experiments using restriction enzyme digested products, which show definitively that the assembly of the DNA fragments occurs *in vivo*. Together, our study along with those mentioned above demonstrate the applicability of *in vivo* cloning in *E*. *coli* DH5α, while the experimental differences provide a basis for balancing the cost and cloning efficiency for user-specific experimental needs.

In the pUC19-*lacZ* experiments, only a few white colonies were observed in both experimental and vector-only transformations, suggesting that non-homologous end joining was extremely rare in the *E*. *coli* DH5α strain. It is therefore somewhat surprising that the fidelity of the multi-fragment assembly (frequency of colonies containing a correctly assembled plasmid among colonies tested) is lower with single antibiotic selection than that observed for single fragment cloning, and the reason for this is not clear. One possible explanation is that the greater variety of sequences at double-strand breaks increases the chance of having unintended end-microhomologies that may be able to recombine [29], forming unwanted products.

Plasmid DNA is commonly transformed into *E*. *coli* rendered competent either by chemical treatment or electroporation, with the latter method generally yielding higher transformation efficiencies, as measured by CFU per amount added DNA. However, our preliminary results indicated that highly competent chemically-prepared cells (~10^9^ CFU/μg pUC19) produce substantially more colonies than electroporated cells (~10^10^ CFU/μg pUC19) in the pUC19-*lacZ* assemblies (Table 1). This result was supported by other assembly transformations attempted in our group and may be consistent with the previously described lack of recombinants using electroporation for DNA assembly in DH5α [25,28]. We hypothesize that the observed difference in assembly efficiency can be explained by the different mechanisms of DNA transport in the two approaches. The conditions employed for the transformation of chemically competent cells are believed to promote the formation of multiple channels per cell as well as DNA crowding at the cell membranes. Uptake of one DNA molecule does not affect uptake of additional DNA, and multiple DNA molecules are commonly introduced into the same cell [31]. On the other hand, in electroporation-induced transformation, the DNA appears to enter the cells in a stochastic manner through the pores formed during the short electrical pulse. At the DNA concentrations employed in our experiments (1–10 ng DNA / ~4×10^10^ cells), not all viable cells are likely to be transformed due to the limited DNA present [37]. Thus, the probability that the same viable competent cell will take up all DNA fragments required for the correct assembly would decrease exponentially with each additional fragment. Based on the observations of Koskela and Frey, [30] it is also possible that in the transformation method of chemically-induced cells, DNA fragments to be assembled begin to interact during the incubation of DNA with competent cells before heat shock, further enhancing the efficiency of this method in comparison to electroporation-induced transformation. *E*. *coli*-mediated *in vivo* assembly methods employing the λ Red and RecET systems rely on electroporation for the co-transformation of DNA fragments to be assembled and usually call for relatively high DNA concentrations of 100 ng or more of each PCR fragment per transformation [16,18,38]. It is not clear whether a similar comparison between highly competent chemically induced cells and electroporation induced cells has been performed in the published experiments.

Many studies have focused on mechanisms of homologous recombination in *E*. *coli*. RecA is the major bacterial recombination protein, essential for repair and maintenance of DNA in the cell [39]. RecA-dependent *in vivo* cloning with linear DNA fragments has been demonstrated in *E*. *coli*, but the assembly of fragments with short end-homology had low efficiency [40]. This was later supported by Lovett et al., showing that RecA-dependent recombination is optimal with homologous regions longer than ~150 bp [22]. The phage-based λ Red and RecET systems are the major mechanisms of RecA-independent homologous recombination that have been studied in *E*. *coli*. Both systems have shown promise for *in vivo* assembly with short regions of homology (<50 bp [18]). The Red system has been studied and employed mainly for engineering of the *E*. *coli* chromosome and BACs, while the RecET system has shown greater utility for *in vivo* assembly of linear fragments [16,17,38]. Established procedures call for specialized strains expressing the Red or RecET systems and recommend a second transformation step into a *recA* ^-^ laboratory strain subsequent to “recombineering” in the specialized strain [18]. Based on current literature, DH5α does not contain an active form of either phage-encoded system. Other mechanisms of RecA-independent recombination have been identified in *E*. *coli*, both for recombination of double- and single-stranded DNA, but have not been fully characterized [22,24]. For double-stranded DNA, RecA-independent mechanisms have been found to be dominant for recombination of short homologous sequences (<50 bp) and to be limited by exonuclease activity [22,23]. We hypothesize that one of these mechanisms enables DH5α to efficiently assemble DNA fragments, as shown here. The observed frequency of recombinants for two-fragment assemblies in DH5α is ~10^−7^ per viable cell (Table 1), which is substantially lower than that for either RecET (~10^−3^–10^−4^) or λ Red (~10^−5^) systems [18]. Nevertheless, as demonstrated here, sufficient colony numbers for many applications are obtained through transformation in highly competent DH5α cells, even for multi-fragment DNA assemblies. The lower recombination efficiency thus becomes an advantage, as it allows the same cell to be used to assemble, clone, and amplify the recombinant DNA.

As with any method, *E*. *coli*-mediated DNA assembly has some limitations. Based on the early Bubeck et al. study [25] and the colony numbers obtained in our experiments, it seems essential to use highly competent cells. While we have not fully explored the size limitation for DNA fragments used for assembly, results obtained with the GSU 1371 knockout construct suggest that the use of fragments smaller than ~350 bp may significantly reduce assembly efficiency. It is also known that transformation efficiency decreases with size [31], and it is likely that assembly of fragments larger than 20–30 kb will be challenging. Furthermore, our results suggest that assembly of more than five fragments is difficult and fidelity decreases with increasing number of fragments. Based on the trends observed in our experiments, it is likely that larger transformation volumes and/or greater amounts of DNA would help with at least some difficult assemblies.

Despite the stated limitations, the method described here should be pertinent to most biological applications that require recombinant DNA. Its power is in its simplicity, as it reduces cloning to two basic steps of DNA preparation and transformation of commercially available *E*. *coli* DH5α, with minimal requirements of reagents and time. It can be readily integrated into a wide range of experimental workflows.

## Supporting Information

S1 Fig pUC19-*LacZα* assembly screen. Following transformation and recovery, cells were plated on LB agar plates containing ampicillin, X-gal, and IPTG. Blue colonies indicate correct assembly of the pUC19 construct. Shown is a plate from an experiment testing the effect of DNA quantity on transformation efficiency, quantified as the number of blue colonies (see Fig 2 and S3 Table). 0.5 ng of linearized pUC19 was used.
(PDF)

S2 Fig Assembly of *Aspergillus niger cbhA* into a custom vector. Fragments shared 50 bp of homology at their ends (see Materials and Methods). A range of vector DNA concentrations was tested while maintaining the insert-to-vector ratio at 5:1. Colony PCR confirmed the presence of the correct insert in 30/30 transformants tested, and Sanger sequencing of 20 junctions confirmed correct assembly in all but one case, where a one-base deletion was identified.
(PDF)

S3 Fig Colony PCR of *A*. *niger cbhA* assembly. Thirty colonies were tested using primers 5-F and 5-R (S2 Table). Expected band size was 1.4 kb.
(PDF)

S4 Fig Colony PCR of *G*. *sulfurreducens* GSU1371 knockout construct assembly, selected on Kan-Amp. Thirty colonies were tested using primers pBR-F and pBR-R (S2 Table). Expected band size was 2.52 kb.
(PDF)

S5 Fig Colony PCR of *G*. *sulfurreducens* GSU1371 knockout construct assembly, selected on Amp only. Thirty five colonies were tested using primers pBR-F and pBR-R (S2 Table). Expected band size was 2.52 kb.
(PDF)

S6 Fig Colony PCR of *C*. *thermocellum cipA* assembly. Ten colonies from each molar ratio experiment (see S6 Table) were tested using primers M13-F(-40) and M13-R (S2 Table). Expected band size was 2.47 kb.
(PDF)

S1 Table High-throughput cloning of cellulase and other carbohydrate-active enzyme genes. (PDF)

S2 Table Primers used for PCR amplification and DNA assembly verification. (PDF)

S3 Table pUC19-*lacZ*α assembly assay. (PDF)

S4 Table *In vivo* assembly of the knockout cassette for gene 1371 in *Geobacter sulfurreducens* from three fragments into a pBR322 vector. (PDF)

S5 Table Comparison of single and double selections on colony number and the fidelity of the GSU 1371 knockout construct. (PDF)

S6 Table *In vivo* assembly of *Clostridium thermocellum cipA* from three fragments into pUC19. (PDF)

S7 Table Effect of fragment number on the assembly of a pBR322-based knockout construct for the deletion of gene GSU 1371 in *Geobacter sulfurreducens*. (PDF)
