# Supplementary figures and images for: Cloning Should Be Simple: Escherichia coli DH5α-Mediated Assembly of Multiple DNA Fragments with Short End Homologies
Source: PLoS One. 2015 Sep 8;10(9):e0137466. doi: 10.1371/journal.pone.0137466 (PMC4562628; doi:10.1371/journal.pone.0137466)

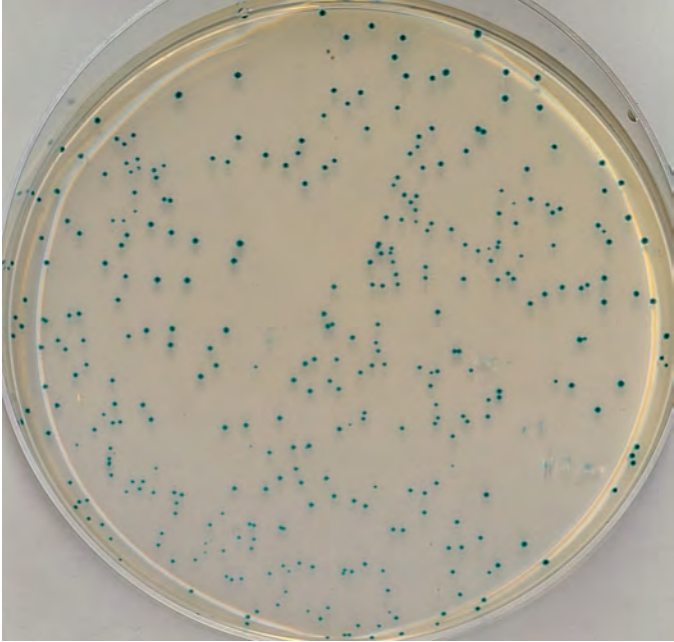

Supplement: S1 Fig — Following transformation and recovery, cells were plated on LB agar plates containing ampicillin, X-gal, and IPTG. Blue colonies indicate correct assembly of the pUC19 construct. Shown is a plate from an experiment testing the effect of DNA quantity on transformation efficiency, quantified as the number of blue colonies (see Fig 2 and S3 Table). 0.5 ng of linearized pUC19 was used. (PDF) [file pone.0137466.s001.pdf]

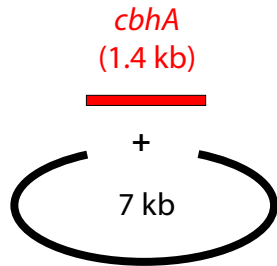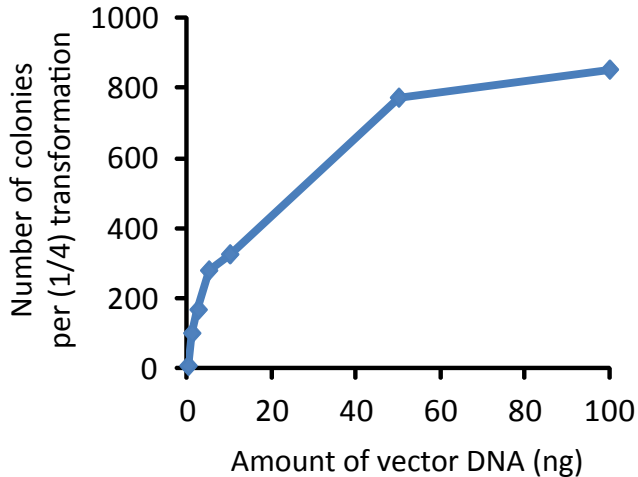

Supplement: S2 Fig — Fragments shared 50 bp of homology at their ends (see Materials and Methods). A range of vector DNA concentrations was tested while maintaining the insert-to-vector ratio at 5:1. Colony PCR confirmed the presence of the correct insert in 30/30 transformants tested, and Sanger sequencing of 20 junctions confirmed correct assembly in all but one case, where a one-base deletion was identified. (PDF) [file pone.0137466.s002.pdf]

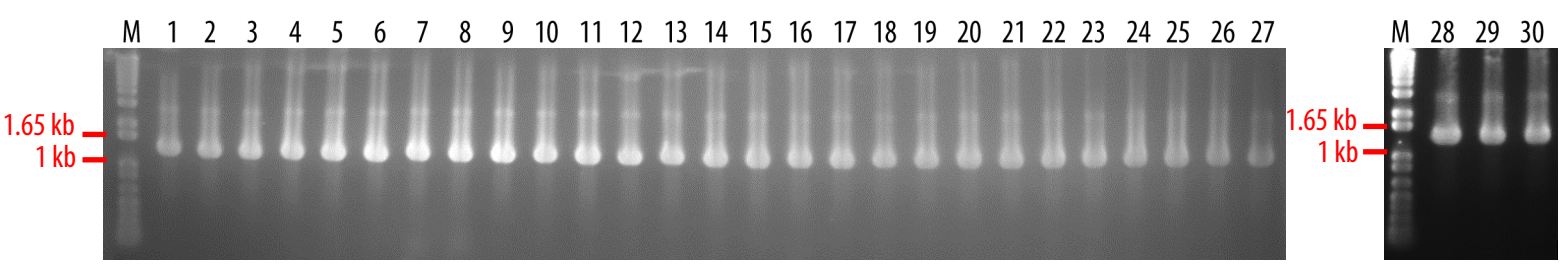

Supplement: S3 Fig — Thirty colonies were tested using primers 5-F and 5-R (S2 Table). Expected band size was 1.4 kb. (PDF) [file pone.0137466.s003.pdf]

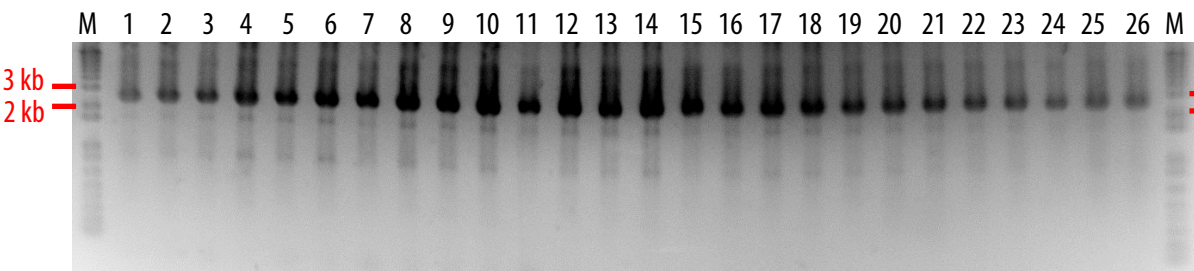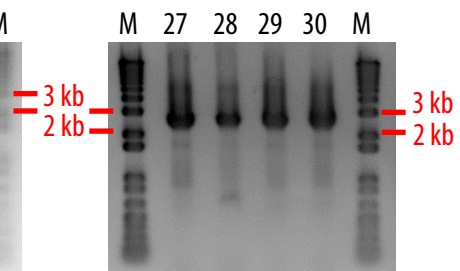

Supplement: S4 Fig — Thirty colonies were tested using primers pBR-F and pBR-R (S2 Table). Expected band size was 2.52 kb. (PDF) [file pone.0137466.s004.pdf]

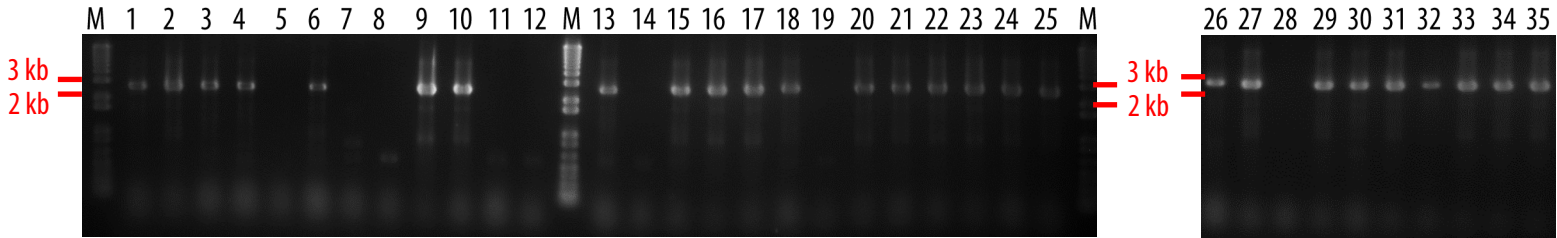

Supplement: S5 Fig — Thirty five colonies were tested using primers pBR-F and pBR-R (S2 Table). Expected band size was 2.52 kb. (PDF) [file pone.0137466.s005.pdf]

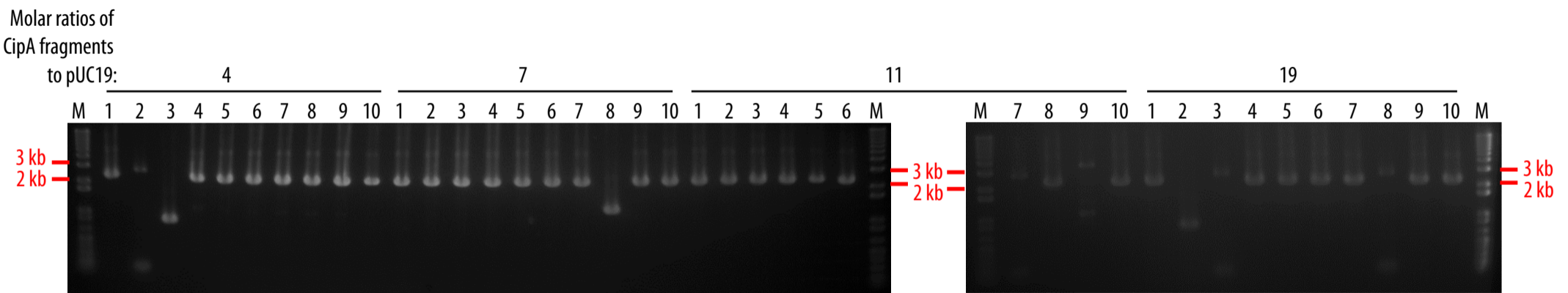

Supplement: S6 Fig — Ten colonies from each molar ratio experiment (see S6 Table) were tested using primers M13-F(-40) and M13-R (S2 Table). Expected band size was 2.47 kb. (PDF) [file pone.0137466.s006.pdf]
