# Supplementary material for: Cloning Should Be Simple: Escherichia coli DH5α-Mediated Assembly of Multiple DNA Fragments with Short End Homologies
Source: PLoS One. 2015 Sep 8;10(9):e0137466. doi: 10.1371/journal.pone.0137466 (PMC4562628; doi:10.1371/journal.pone.0137466)
Supplement: S1 Table — (PDF) [file pone.0137466.s007.pdf]

S1 Table. High-throughput cloning of cellulase and other carbohydrate-active enzyme genes.

Reaction ID	Species	Family	Fragment size (bp)[a]	Colonies per transformation[b]	Correct band in colony PCR	Issues with PCR?
1	*Talaromyces emersonii*	GH6	1411	18	2/3	multiple bands
2	*Neurospora crassa*	AA9	769	45	3/3	smear
3	*Thermobifida fusca*	AA10	658	31	1/1	-
4	*Chrysosporium lucknowense*	GH7	1282	54	2/3	multiple bands
5	*Acidothermus cellulolyticus*	GH12	769	100	1/1	-
6	*Chaetomium thermophilum*	GH7	1456	35	1/3	multiple bands
7	*Neosartorya fischeri*	GH7	1456	43	1/1	multiple bands
8	*Clostridium termitidis*	GH8	1216	56	1/1	-
9	*Gibberella zeae*	GH45	1003	125	1/1	-
10	*Trichoderma viride*	GH5	1357	130	1/1	-
11	*Paenibacillus barcinonensis*	GH5	1201	112	1/1	-
12	*Talaromyces stipitatus*	GH5	1063	87	1/1	multiple bands
13	*Talaromyces cellulolyticus*	GH6	1201	69	3/3	multiple bands
14	*Talaromyces marneffei*	GH7	1435	65	1/1	-
15	*Saccharophagus degradans*	GH5	943	42	1/1	-
16	Uncultured organism	GH5	1024	80	1/1	-
17	*Aureobasidium pullulans*	GH5	1000	24	2/2	multiple bands
18	*Trichoderma koningii*	GH6	1162	14	2/2	-
19	*Cytophaga hutchinsonii*	GH5	913	30	2/2	-
20	*Trichoderma koningii*	GH12	754	11	2/2	-
21	Uncultured organism	GH5	997	5	2/2	-
22	*Thielavia terrestris*	GH7	1459	5	1/2	multiple bands
23	*Myceliophthora thermophila*	GH5	985	16	2/2	-
24	*Thielavia terrestris*	GH1	1525	16	2/2	-
25	*Thielavia terrestris*	GH6	1189	25	2/2	-
26	*Trichoderma reesei* [c]	GH6	1447	8	1/2	multiple bands
27	*Trichoderma reesei*	GH6	1447	19	2/2	-
28	*Trichoderma reesei* [c]	GH5	1297	24	3/3	-
29	*Trichoderma reesei*	GH5	1297	4	2/3	-

[a] All fragments contained 40 bp of overlapping sequences with the vector at each end; coding sequences were codon-optimized using a published algorithm (Lanza et al., 2014), synthesized with BioXp™ 3200 (SGI-DNA), and PCR-amplified with PrimeSTAR Max polymerase (Takara Bio).

[b] 5 ng of a 7-kb custom vector was cotransformed with 5 ng insert, giving molar insert-to-vector ratio between 5:1 and 9:1.

[c] Native rather than codon-optimized cellulase sequences were used in these reactions.
