# Supplementary material for: Cloning Should Be Simple: Escherichia coli DH5α-Mediated Assembly of Multiple DNA Fragments with Short End Homologies
Source: PLoS One. 2015 Sep 8;10(9):e0137466. doi: 10.1371/journal.pone.0137466 (PMC4562628; doi:10.1371/journal.pone.0137466)
Supplement: S2 Table — (PDF) [file pone.0137466.s008.pdf]

S2 Table. Primers used for PCR amplification and DNA assembly verification.

Fragment	Primer name	Primer sequence	Template	Final construct
vector (LacZ)	1-F	TTATAGGTTAATGTCATGATAATAATGGTTTCTTAGAC	pUC19	pUC19 (reassembled)
	1-R	GGATCCTCTAGAGTCGACCTG		
LacZ	2-F	CCATGATTACGCCAAGCTTGCATG	pUC19	
	2-R	GTGCCACCTGACGTCTAAGAAACC		
vector (AnCbhA)	3-F	ATTCAACTTTCAGTGCTTCTGCGGGTGAAGCTCCAAAAGTTACCAC	pYOGM081-Cel	pYOGM081-Cel-AnCbhA
	3-R	TAAGAGTACCGGCTTGCTGAGAGCCACCTCCACCGGAAC		
AnCbhA	3-F	AGGAGGTGGCGGTTCCGGTGGAGGTGGCTCTCAGCAAG	synthetic DNA (BioXP™ 3200)	
	3-R	TGTAGTGGTAACTTTTGGAGCTTCAC		
vector (cellulase)	4-F	CAAAAGTTACCACTACAACAACTTCCTCTG	pYOGM081-Cel	pYOGM081-Cel-Cellulase
	4-R	CCACCGGAACCGCCACCTCCTGAG		
Cellulase	5-F	CTTCAGGAGGGGGTGGCTC	synthetic DNA (BioXP™ 3200)	
	5-R	GTTGTGACGGCAGAGGAAG		
Kan-cassette	6-F	ATGAACCACCAGCCAAGGAGGCACGCCGTATCTCAGTTCGGTGTAGGTCG	pET28a	All KO constructs
	6-R	TAGTTTCGCGTGCGGTTCACGGCTACAATTTCAGGTGGCACTTTTCG		
vector-KO-1150	7-F	GAATTCTGCAGCAAAGCCATGGAAACGGTGGGATCCTCTACGCCGGACGCA	pBR322	KO-1150
	7-R	TGCCGAAATAGTGCCTGATCCTGTCGAAAGACGAAAGGGCCTCGTGATACG		
5'-1150	8-F	GTATCACGAGGCCCTTTCGTCTTTCGACAGGATCAGGCACTATTTCG	*G. sulfurreducens* gDNA	
	8-R	GAACGACCTACACCGAACTGAGATACGGCGTGCCTCCTTGGCTGGTG		
3'-1150	9-F	TTTCCCCGAAAAGTGCCACCTGAAATTGTAGCCGTGAACCGCACGCGAAAC	*G. sulfurreducens gDNA*	
	9-R	ACGATGCGTCCGGCGTAGAGGATCCCACCGTTTCCATGGCTTTGC		
vector-KO-550	10-F	AGCTGCACCAGATCGCCCAGGAAAAGGACTGGATCCTCTACGCCGGACGCA	pBR323	KO-550
	10-R	TCGCCCAGGTGGAGGCATTTCTTGCGAAAGACGAAAGGGCCTCGTGATACG		
5'-550	11-F	GTATCACGAGGCCCTTTCGTCTTTCGCAAGAAATGCCTCCACCTGGGCGAG	*G. sulfurreducens* gDNA	
	8-R	GAACGACCTACACCGAACTGAGATACGGCGTGCCTCCTTGGCTGGTG		
3'-550	9-F	TTTCCCCGAAAAGTGCCACCTGAAATTGTAGCCGTGAACCGCACGCGAAAC	*G. sulfurreducens* gDNA	
	12-R	ACGATGCGTCCGGCGTAGAGGATCCAGTCCTTTTCCTGGGCGATCTGG		
vector-KO-350	13-F	CCGGGGGATCACCTTCTTCGACACGGGATCCTCTACGCCGGACGCA	pBR323	KO-350
	13-R	GGGAATTCACCGCTCAGGAGCATCTGAAAGACGAAAGGGCCTCGTGATACG		
5'-350	14-F	GTATCACGAGGCCCTTTCGTCTTTCAGATGCTCCTGAGCGGTGAATTCC	*G. sulfurreducens* gDNA	
	8-R	GAACGACCTACACCGAACTGAGATACGGCGTGCCTCCTTGGCTGGTG		
3'-350	9-F	TTTCCCCGAAAAGTGCCACCTGAAATTGTAGCCGTGAACCGCACGCGAAAC	*G. sulfurreducens* gDNA	
	15-R	ACGATGCGTCCGGCGTAGAGGATCCCGTGTCGAAGAAGGTGATCC		
vector-KO-250	16-F	CTCGGTTCGACCGGGCTGACCGTTTGGATCCTCTACGCCGGACGCA	pBR324	KO-250
	16-R	TCGTTGGCCGTGGCCGCGCCGTGGAGAAAGACGAAAGGGCCTCGTGATACG		
5'-250	17-F	GTATCACGAGGCCCTTTCGTCTTTCTCCACGGCGCGGCCACGGCCAAC	*G. sulfurreducens* gDNA	
	8-R	GAACGACCTACACCGAACTGAGATACGGCGTGCCTCCTTGGCTGGTG		
3'-250	9-F	TTTCCCCGAAAAGTGCCACCTGAAATTGTAGCCGTGAACCGCACGCGAAAC	*G. sulfurreducens* gDNA	
	18-R	ACGATGCGTCCGGCGTAGAGGATCCAAACGGTCAGCCCGGTCGAAC		
vector-KO-150	19-F	CGGGTGCCGGGCGTTCAGCCCTCCTCGCCAGGATCCTCTACGCCGGACGCA	pBR325	KO-150
	19-R	CCCCGGCAGGAATGGCGCTACTCCAGAAAGACGAAAGGGCCTCGTGATACG		
5'-150	20-F	GTATCACGAGGCCCTTTCGTCTTTCTGGAGTAGCGCCATTCCTGC	*G. sulfurreducens* gDNA	
	8-R	GAACGACCTACACCGAACTGAGATACGGCGTGCCTCCTTGGCTGGTG		
3'-150	9-F	TTTCCCCGAAAAGTGCCACCTGAAATTGTAGCCGTGAACCGCACGCGAAAC	*G. sulfurreducens* gDNA	
	21-R	GAACGACCTACACCGAACTGAGATACGGCGTGCCTCCTTGGCTGGTG		
vector-CipA	22-F	ACAATAAGCGAATTTCTTATGATTTATGGATCCTCTAGAGTCGACCTGC	pUC19	pUC19-CtCipA
	22-R	GAGCCACCCCCTCCTGAAGCCGGGTACCGAGCTCGAATTC		
CipA-F1	23-F	GACGGCCAGTGAATTCGAGCTCGGTACCCGGCTTCAGGAGGGGGTGGCTCAG	synthetic DNA (gBlocks®, IDT)	
	23-R	CGTAGGGGTCGCGCCCTTAG		
CipA-F2	24-F	GTGGAGTAAACGTTGGAAATGCAAC	synthetic DNA (gBlocks®, IDT)	
	24-R	TTTTTAGCAAAACGTCCCTGAATCTG		
CipA-F3	25-F	ACTTTAGAACCTGGAGCTCACGTG	synthetic DNA (gBlocks®, IDT)	
	25-R	TGCAGGTCGACTCTAGAGGATCCATAAATCATAAGAAATTCGCTTATTGTGC		
p426-gRNA-GMTK-KO	26-F	TCGCCCTTGGAATTGACGAGTAGTTTTAGAGCTAGAAATAGCAAG	p426-SNR52p-gRNA-CAN1.Y-SUP4t	p426-gRNA-GMTK-KO
	26-R	AACTACTCGTCAATTCCAAGGGCGATCATTTATCTTTCACTGCGGAG		
p426-gRNA-YGR176W	27-F	TCCTCGTAGTCGATGCATGCCGGTTTTAGAGCTAGAAATAGCAAG	p426-SNR52p-gRNA-CAN1.Y-SUP4t	p426-gRNA-YGR176W
	27-R	AACCGGCATGCATCGACTACGAGGATCATTTATCTTTCACTGCGGAG		
P_{TRC}_gRNA_pUC-FEPC	28-F	GTAAAGAGGTTGCTCGCCGCCGGTGTGTGTGAAATTGTTATCC	P_{TRC}_gRNA_pUC	P_{TRC}_gRNA_ pUC-FEPC
	28-R	GCGGCGAGCAACCTCTTTACGTTTTAGAGCTAGAAATAGCAAGTTAAAATAAGG		
Sequencing / verification	M13-F(-40)	GTTTTCCCAGTCACGAC	pUC19-based and p426-SNR52p-gRNA-based constructs	N/A
	M13-R	CAGGAAACAGCTATGAC		
	pBR-F	AAAGTGCCACCTGACGTCTAAG	pBR322-based constructs	
	pBR-R	ATCTTCCCCATCGGTGATGTCG		

	5-F	CTTCAGGAGGGGGTGGCTC	pYOGM081-Cel-AnCbhA	
	5-R	GTTGTGACGGCAGAGGAAG		
	Cell-F	CTTGTAATCCCTTATTCCTTCTAGC	Cellulase constructs (except pYOGM081-Cel-AnCbhA)	
	Cell-R	CATCTGGGCAGATGATGTCGAG		
	Seq_CR	CTCTCATCCGCCAAAACAGC	P_{TRC}_gRNA_pUC-FEPC	
