# Supplementary material for: Cloning Should Be Simple: Escherichia coli DH5α-Mediated Assembly of Multiple DNA Fragments with Short End Homologies
Source: PLoS One. 2015 Sep 8;10(9):e0137466. doi: 10.1371/journal.pone.0137466 (PMC4562628; doi:10.1371/journal.pone.0137466)
Supplement: S3 Table — (PDF) [file pone.0137466.s009.pdf]

S3 Table. pUC19-*lacZ* α assembly assay.

Vector added (ng)	Insert added (ng)[a]	Blue colonies[b]	White colonies
0.1	0.12	24	0
0.2	0.24	98	0
0.5	0.6	340	3
1	1.2	896	4
2	2.4	1220	2
5	6	1778	5
10	12	2040	1
0.1	0	0	0
0.2	0	0	1
0.5	0	0	1
1	0	4	1
2	0	1	1
5	0	2	2
10	0	6	2
0	6	5	0
0	12	10	0

[a] The insert-to-vector molar ratio was 5:1 for all reactions.

[b] Shown are colony numbers per 25 µl cells, which corresponds to 1/4 recommended transformation cell volume.
