# Supplementary material for: Cloning Should Be Simple: Escherichia coli DH5α-Mediated Assembly of Multiple DNA Fragments with Short End Homologies
Source: PLoS One. 2015 Sep 8;10(9):e0137466. doi: 10.1371/journal.pone.0137466 (PMC4562628; doi:10.1371/journal.pone.0137466)
Supplement: S4 Table — (PDF) [file pone.0137466.s010.pdf]

S4 Table. ***In vivo*** **assembly of the knockout cassette for gene 1371 in *Geobacter sulfurreducens* from three fragments into a pBR322 vector.**

Vector added (ng)[a]	5' target sequence added (ng)	Kanamycin cassette added (ng)[a]	3' target sequence added (ng)	Colonies on Kan-Amp	Colonies on Kan[b]	Colonies on Amp[b]
1	0.68	1.8	0.68	15	ND	ND
5	3.4	9.1	3.4	65	ND	ND
10	6.8	18	6.8	92	ND	ND
50	34	91	34	177	ND	ND
100	68	182	68	204	ND	ND
10[c]	0	18	0	0	93	556

[a] DpnI digest after PCR was omitted because the contaminating template plasmid with only the Amp marker or the Kan marker is unable to produce colonies in double selection.

[b] ND = Not determined

[c] A control sample lacking fragments for bridging the fragments carrying antibiotics resistance markers confirmed that no colony is generated with contaminating template plasmids in double selection in the absence of correct assembly.
