# Supplementary material for: Cloning Should Be Simple: Escherichia coli DH5α-Mediated Assembly of Multiple DNA Fragments with Short End Homologies
Source: PLoS One. 2015 Sep 8;10(9):e0137466. doi: 10.1371/journal.pone.0137466 (PMC4562628; doi:10.1371/journal.pone.0137466)
Supplement: S5 Table — (PDF) [file pone.0137466.s011.pdf]

S5 Table. Comparison of single and double selections on colony number and the fidelity of the GSU 1371 knockout construct.

Sample	Selection	Colonies per transformation	Correct band in colony PCR
Vector only[a]	Amp	4	N/A
Assembly[b]	Amp/Kan	61	10/10
Assembly[b]	Amp	126	27/35
Assembly[b]	Kan[c]	68	10/10

[a] Only the vector fragment (10 ng) was introduced into 25 µl of cells as a negative control.
[b] The insert DNA fragments were first combined with 10 ng of the vector fragment at a 5:1 molar ratio for insert and vector, and then with 25 µl of cells.
[c] Although Kan was the only antibiotic used, this selection was equivalent to double selection because the origin of replication needed in the final product was provided by the vector fragment.
