# Supplementary material for: Cloning Should Be Simple: Escherichia coli DH5α-Mediated Assembly of Multiple DNA Fragments with Short End Homologies
Source: PLoS One. 2015 Sep 8;10(9):e0137466. doi: 10.1371/journal.pone.0137466 (PMC4562628; doi:10.1371/journal.pone.0137466)
Supplement: S6 Table — (PDF) [file pone.0137466.s012.pdf]

S6 Table. ***In vivo* assembly of *Clostridium thermocellum cipA* from three fragments into pUC19.**

Molar ratio of *cipA* fragments to pUC 19[a]	Colonies per transformation[b]	Correct band in colony PCR
4	66	8/10
7	96	9/10
11	95	8/10
19	39	7/10
0	4	N/A

[a] *cipA* fragments were 996, 499, and 1009 bp.
[b] 10 ng of vector was used in all transformations.
