# Supplementary material for: Cloning Should Be Simple: Escherichia coli DH5α-Mediated Assembly of Multiple DNA Fragments with Short End Homologies
Source: PLoS One. 2015 Sep 8;10(9):e0137466. doi: 10.1371/journal.pone.0137466 (PMC4562628; doi:10.1371/journal.pone.0137466)
Supplement: S7 Table — (PDF) [file pone.0137466.s013.pdf]

S7 Table. Effect of fragment number on the assembly of a pBR322-based knockout construct for the deletion of gene GSU 1371 in *Geobacter sulfurreducens.*

	Kan-Amp selection		Amp selection	
Number of fragments[a]	Colonies per transformation[b]	Correct band in colony PCR	Colonies per transformation[b]	Correct band in colony PCR
5	10	5/5	19	5/10
6	4	4/4	14	6/8

[a] Fragment sizes were 551, 1,466, 555, 3,320, and 765 bp for five-fragment assembly; and 551, 1,466, 555, 1,803, 1,567, and 765 bp for six-fragment assembly.

[b] Fragments were added at an equimolar ratio to the collective amount of ~125 ng per 25 µl transformation.
